# Syntactic Complexity Development in CSL Writing: A Perspective from Dynamic Systems Theory

**DOI:** 10.3390/bs16040590

**Published:** 2026-04-15

**Authors:** Huan Zhang

**Affiliations:** 1 College of Humanities and Law, South China Agricultural University, Guangzhou 510642, China; huan0914@scut.edu.cn; 2 College of Chinese Language and Culture, Jinan University, Guangzhou 510610, China

**Keywords:** CSL writing, syntactic complexity, Dynamic Systems Theory

## Abstract

Syntactic complexity is a crucial aspect of assessing writing quality in Chinese as a second language. While existing literature predominantly focuses on synchronic features of syntactic complexity, particularly changes in complexity indices, less attention has been paid to its diachronic development and interactions among these indices. Drawing upon Dynamic Systems Theory, this exploratory longitudinal study traces the developmental trajectories of syntactic complexity indices and their interactions in CSL writings of 15 native Cambodian speakers within a single instructional context. The main results are as follows: (i) The syntactic complexity indices exhibited fluctuating and nonlinear growth. Among them, the length of topic chain clauses showed notable variation (range: 1.12 to 9.05), while relatively small changes (range: 0.4 to 2.39) occurred in the number of topic chain clauses. (ii) The development trends in the number of topic chains and the number of zero components showed no significant difference (*p* = 0.086, Cohen’s d = 0.31). In contrast, the development trends in the number of topic chain clauses and the length of topic chain clauses differed significantly (*p* = 0.039, Cohen’s d = 0.65). (iii) Individual differences in syntactic complexity were observed among learners in similar learning environments. These findings provide a detailed, context-bound description of the dynamic and complex syntactic development observed in the Chinese writing of 15 participants. The study highlights the value of employing multiple perspectives to capture such complexity and underscores the need for future research with more diverse samples and designs to test the generalizability of these patterns.

## 1. Introduction

With the advancement of research, syntactic complexity, widely acknowledged as a key factor in assessing writing quality, has garnered increasing attention in the field of second language (L2) writing. In recent decades, there has been a growing body of research focusing on syntactic complexity in L2 writing (e.g., Jin, 2007; Norris & Ortega, 2009; Verspoor et al., 2012; Qi & Liao, 2019; H. Zhang & Wang, 2022; Hwang & Kim, 2024), yielding numerous achievements. However, as research delves deeper, it reveals that the development of syntactic complexity is not continuous or linear, but rather dynamic and variable. Therefore, the static research paradigm must be challenged to encourage researchers to reexamine the entire process of syntactic acquisition. In this context, Dynamic Systems Theory (DST) has emerged as a promising framework for conducting research in this area.

The research on the syntactic complexity of L2 writing based on DST was initially conducted in the field of English as a Second Language (ESL). These findings suggest that DST can effectively explain various complex language phenomena observed during L2 syntactic acquisition, including non-linearity, variation, erosion, regression and progress (e.g., Larsen-Freeman, 2006; Ji, 2009; Wang et al., 2015; Huang & Zheng, 2022). Due to its stronger explanatory power, researchers have introduced it to Chinese as a Second Language (CSL) and carried out related empirical studies (Wu, 2017; L. Zhou, 2020). However, there has been relatively little research on Chinese syntactic complexity based on DST overall. Existing studies mainly focus on changes in the syntactic complexity of individual Chinese L2 learners and lack further analysis of potential relationships among these indices. This results in an incomplete understanding of the developmental process of Chinese syntactic complexity. Therefore, this study adopts DST as its theoretical framework to examine the dynamic changes and features of Chinese syntactic complexity in writings by 15 CSL learners. It aims to reveal correlations and interactions among various indices, as well as to offer pedagogical implications.

## 2. Literature Review

### 2.1. Syntactic Complexity

Syntactic complexity refers to the range of variation and the degree of structural complexity in syntactic structures used in productive language (Ortega, 2003; X. Lu, 2011; Bulté & Housen, 2014). Currently, research on syntactic complexity in CSL is becoming increasingly detailed, and related achievements can be broadly categorized into three types.

The first type involves the macroscopic construction of a measurement system for syntactic complexity in L2 acquisition and the exploration of effective dimensions and indices (e.g., Jin, 2007; Yuan, 2009; Norris & Ortega, 2009; Verspoor et al., 2012; Jiang, 2012; Y. Zhang et al., 2015; X. Lu & Xu, 2016). Jin (2007) and Yuan (2009) argued that the T-unit was not an effective measure of Chinese syntactic complexity. However, Jiang (2012) found that the percentage of error-free T-unit was relatively reliable. Thus, the validity of using T-unit to measure Chinese syntactic development remains controversial and requires further investigations to demonstrate its effectiveness. Moreover, Jin (2007) incorporated topic chains and zero components as measures of Chinese syntactic complexity, which Wu (2016) empirically validated as effective dimensions. The second type investigates the developmental patterns of syntactic complexity in L2 acquisition at a microscopic level (e.g., Ellis & Yuan, 2004; Zhao & Chen, 2012; Qi & Liao, 2019; W. Zhou, 2022). Among them, H. Zhang and Wang (2022) conducted a related study on syntactic complexity in CSL writing, using Cambodian native speakers as participants. They identified two developmental stages: the stage of primary topic-and-subject mixed prominence and the stage of topic-prominence. This study represented the first attempt to specifically examine syntactic complexity in CSL writing among Cambodian native speakers. The third type relates to the factors influencing syntactic complexity in L2 writing (Way et al., 2000; Ortega, 2003; Pei, 2020; Wang et al., 2022). Way et al. (2000) and L. Lu and Sun (2012) investigated the impact of different writing tasks. The former found that descriptive tasks were easier, while expository tasks were more difficult. The latter revealed a significant influence of communicative task types on syntactic complexity. Ortega (2003) and Pei (2020) reported that the learning context played a role in shaping the relationship between L2 proficiency and syntactic complexity in writing.

The above studies at home and abroad mainly focus on ESL, discussing the synchronic features of syntactic complexity in writing. However, relatively less attention has been paid to the dynamic changes in syntactic complexity in CSL writing, leading to certain limitations. In fact, Chinese syntactic complexity is not simply an approximately linear continuum; instead, it exhibits variation. Investigating its developmental process facilitates a comprehensive understanding of syntax acquisition as a whole. Therefore, further examination of this procedural ‘black box’ is necessary.

### 2.2. Dynamic Systems Theory

The Dynamic Systems Theory (DST) originated as a rigorous mathematical framework for analyzing complex systems over time. Larsen-Freeman (1997) introduced DST into applied linguistics to investigate language as a complex adaptive system. Li (2011) argued that DST had great significance in that it not only challenged traditional Reductionism, directly addressing the complex and dynamic process of L2 learning, but also developed corresponding research methods and tools, making an important contribution. The influence of DST is gradually shaping new perspectives on language, which can be understood from three aspects. (i) DST views language as a self-organizing and self-adaptive system composed of interconnected subsystems that mutually constrain one another (De Bot, 2008; Larsen-Freeman, 2017; Zheng, 2019). (ii) DST believes that language development is a multifaceted and nonlinear process characterized by variability, including progress, regression and stagnation, which is difficult to predict (Larsen-Freeman, 2009; Verspoor & Van Dijk, 2013). (iii) DST emphasizes the importance of paying greater attention to individual differences in language acquisition. Similarly, syntactic development exhibits noticeable dynamics and complexity.

Recent years have witnessed a growing number of empirical studies on syntactic complexity based on DST, primarily focusing on ESL. The existing research achievements can be grouped into three thematic strands. First, one strand involves the development of syntactic complexity in L2 writing, investigating longitudinal changes and the ways in which indices interact. Jiang and Wang (2015) conducted related empirical research and argued that syntactic development is dynamic and variable, with a deep interactive relationship among syntactic dimensions, including competition and support. Secondly, another strand focuses on the interaction between syntax and other language subsystems, revealing changes in overall language quality (e.g., Verspoor et al., 2008; Spoelman & Verspoor, 2010; Wang et al., 2015; Wu, 2017; Zheng & Feng, 2017; Zheng, 2018). These studies emphasize that language development is a complex and nonlinear process in which linguistic subsystems interact across multiple levels. Thirdly, a third strand concentrates on individual differences in the syntactic developmental pattern of L2 learners. Chan et al. (2015) and Yu et al. (2022) reported significant individual and cross-population differences in syntactic development, indicating that syntactic development does not follow a uniform trajectory but rather exhibits population-specific characteristics.

On the whole, most of the research above focuses on ESL learning; however, relatively little attention has been paid to how syntactic complexity in CSL writing changes over time and how these indices interact. With the growing international prominence of the Chinese language, an increasing number of foreign friends are choosing it as a second language, making it necessary to investigate Chinese syntactic complexity from a dynamic perspective. In fact, CSL learning is also a complex process characterized by considerable variation. Investigating the dynamic and diachronic features of CSL syntactic complexity holds great significance for assessing language quality and gaining a deeper understanding of L2 acquisition mechanisms. Therefore, further research is needed to broaden theoretical perspectives and methodological approaches. Accordingly, drawing on the DST principle that language subsystems compete for limited cognitive resources (Larsen-Freeman, 2006), we anticipated that indices serving similar linguistic functions might exhibit supportive relationships, while indices tapping different levels of syntactic processing might exhibit competitive relationships. These exploratory expectations provide a theoretical framework for interpreting the developmental patterns observed in this study.

Against this background, this study addresses two main questions:

(1) How does syntactic complexity develop in CSL writing over the course of a school year? Specifically, what variations are observed in syntactic complexity indices?
(2) What are the relationships among Chinese learners’ syntactic complexity indices during development?

## 3. Methodology

This study is based on DST and employs a longitudinal design with 15 CSL learners. The primary focus is on examining syntactic complexity in Chinese writing from the dimensions of topic chain and zero component. The techniques of moving min-max graphs, Monte Carlo simulations, and moving correlation coefficient graphs were utilized to analyze the data, visualizing the dynamic development process of syntactic complexity indices. The first-hand data collected from writings enable us to observe and comprehend the developing trend of syntactic complexity of CSL learners and to gain an in-depth understanding of the correlation and interaction among these indices.

### 3.1. Participants

This empirical study was conducted at a Chinese-language school in Phnom Penh, Cambodia. Participants consisted of 15 Cambodian-speaking CSL learners (9 female, 6 male), aged 13 to 16 years. All participants began learning Chinese in the same year and had taken comparable courses, including Chinese language, Chinese-Cambodian translation, and mathematics. They had received four years of Chinese writing instruction (45 min per week) and had no prior experience studying or residing in Chinese-speaking regions. In other words, they shared a similar educational background.^1^ Prior to the study, participants had mastered approximately 2400 Chinese words and about 60% of core Chinese grammar points. To assess participants’ initial Chinese proficiency, a pretest was administered before the start of the study. The test consisted of a vocabulary and grammar section comprising 30 multiple-choice items (15 vocabulary items, 15 grammar items), adapted from HSK Level 4 practice papers. The test was scored by assigning two points for each correct answer (total score range: 0 to 60). The mean score was 52.29 (SD = 3.16, range: 48 to 60), indicating that participants were relatively homogeneous in Chinese vocabulary and grammar knowledge prior to the study. This performance level corresponds roughly to HSK Level 4 proficiency according to the HSK test specifications.

### 3.2. Materials

This study was conducted in a mandatory Chinese writing course, which lasted 18 weeks per semester and a total of 36 weeks per year. The author served as both the teacher and researcher.

The research materials consist of compositions written by the 15 participants. Participants were scheduled to complete a writing task once every two weeks, totaling eight times per semester and 16 tasks over the entire school year. The writing tasks primarily focus on narrative compositions, covering topics such as friendship, family, job, etc. Each participant was required to complete and submit their composition in class within a 50 min time limit and under the teacher’s supervision. A minimum of 300 characters was required for each composition, and participants were not permitted to use any reference materials or AI-assisted tools during the task. In total, 240 compositions were collected, comprising 97,320 characters (see Table 1).

To assess the comparability of the 16 writing tasks, two experienced Chinese teachers (not involved in the study) independently rated the difficulty of each task on a 5-point Likert scale (1 = very easy, 5 = very difficult). The mean difficulty ratings ranged from 3.25 to 3.31 across tasks. A paired-samples *t*-test showed no significant difference between the two raters (mean difference = −0.06, *t*(15) = −0.565, *p* = 0.580), indicating acceptable inter-rater consistency. These results suggest that the writing tasks were generally comparable in difficulty, although task-specific content differences cannot be entirely ruled out.

### 3.3. Procedure

**Figure 1:**
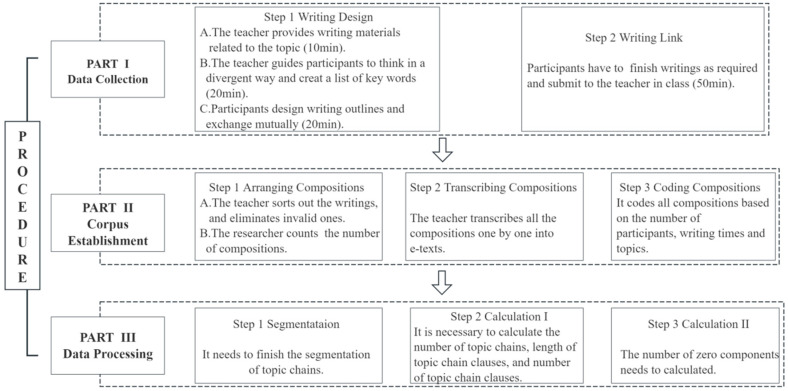
The design of this study.

This study was conducted over one school year, and the research procedure included three parts: data collection, corpus establishment and data processing. The first part involved teaching session in which all participants were required to complete writing tasks. It is important to note that 16 writing tasks were completed in the same manner, once every two weeks. The second part focused on collating the compositions into e-texts to construct a mini-corpus. Lastly, the third part involved corpus processing. Please refer to Figure 1 for a detailed illustration of the entire study.

All compositions were coded by the researcher using the explicit operational definitions of topic chains and zero components outlined in Section 3.4. The coding scheme was applied systematically across all 240 texts. After the initial coding, the researcher conducted two rounds of review to check the accuracy and consistency of all identifications. To assess inter-rater reliability, a random sample comprising 20% of the compositions (n = 48) was independently coded by a second researcher—a Chinese language teacher with six years of teaching experience who was blinded to the research hypotheses and had not participated in the instruction or data collection. Inter-rater reliability was high, with Cohen’s κ values for all indices ranging from 0.78 to 0.84, indicating consistent and reliable application of the coding scheme. Moreover, to assess intra-rater reliability, the same 48 texts were re-coded by the same researcher, yielding an agreement rate of 86%, indicating high internal consistency.

### 3.4. Measurement Dimensions and Indices

Some literature argues that the use of T-units may not be suitable for measuring Chinese syntactic complexity (e.g., Jin, 2007; Yuan, 2009; Wu, 2016). A distinctive feature of the Chinese language is its emphasis on topic prominence and discourse cohesion. Therefore, excluding T-units, this study primarily investigates the dynamic development of Chinese syntactic complexity along two dimensions: topic chains and zero components.

#### 3.4.1. Topic Chain

The term “topic chain” refers to a linguistic unit comprising at least two clauses that share the same topic, which is mentioned only in the first clause and replaced by zero topic in subsequent clauses (Jin, 2007). In this study, the following indices were operationalized: (1) Number of topic chains: the total frequency of topic chains identified in each composition; (2) Length of topic chain clauses: the average length of all clauses in each composition, measured in Chinese characters (excluding punctuation marks); (3) Number of topic chain clauses: the average number of clauses contained in per topic chain. Clause boundaries were identified based on the presence of a predicate (i.e., verb or adjective), following the criteria used in previous CSL studies (e.g., Wu, 2016). In cases of ambiguous segmentation, the minimal clause unit containing a single predicate was used.

#### 3.4.2. Zero Component

The term “zero component” refers to the omission of a topic, subject, or object that is recoverable from the discourse context, as exemplified by the symbol ø. In this study, the number of zero components was operationalized as the total frequency of such omissions in each composition. The following criteria were applied. (1) Only grammatically obligatory and contextually recoverable omissions were counted. Optional pronoun drops were excluded, unless their insertion would render the sentence ungrammatical. (2) Ellipsis was counted only when the omitted element was unambiguously recoverable from the same topic chain. (3) Each omission was counted separately (e.g., three zero-subject clauses = three zero components).

#### 3.4.3. Annotated Examples from Learners’ Data

To illustrate how the coding scheme was applied to participants’ authentic compositions, three examples from the corpus are presented below, along with detailed coding decisions (see Table 2).

### 3.5. Data Analysis Tools

#### 3.5.1. Moving Min-Max Graph

Van Geert and Van Dijk (2002) developed the moving min-max graph, which was initially applied in research on children’s native language acquisition. Verspoor et al. (2008) introduced this method into L2 acquisition research. The graphing procedure involves two steps. First, the data are segmented into multiple overlapping moving windows based on measurement time points. Second, the maximum and minimum values within each window are calculated, generating two series for plotting.

This study comprises 16 writing tasks, yielding a total of 16 measurements. Every four consecutive measurements constitute a sub-series. The algorithm calculates the minimum and maximum values within the first four data points, then shifts the window by one data point to compute the next sub-series, and so on. In total, this moving-window approach generates 13 sub-series.

max(t1…t4), max(t2…t5), … max(t13…t16).

min(t1…t4), min(t2…t5), … min(t13…t16).

#### 3.5.2. Moving Correlation Coefficient

The moving correlation coefficient reflects dynamic changes in the relationship between two time series. The plotting guidelines for this graph are similar to those of a moving min-max plot. By analyzing the correlation coefficients obtained at successive time points, we can construct moving correlation coefficient plots, enabling observation of fluctuations across different phases in these coefficients.

#### 3.5.3. Monte Carlo Simulation

To assess whether the developmental trajectories of different syntactic complexity indices differed significantly, a Monte Carlo simulation with 5000 iterations was conducted following the procedure described in Verspoor et al. (2011). The procedure was as follows: (i) For each index pair (e.g., number of topic chains vs. number of zero components), the observed time series were randomly shuffled with replacement to simulate the null hypothesis that any observed differences are due to random fluctuation. (ii) For each resampled dataset, the mean absolute difference between the two trajectories across all time points was calculated, yielding a null distribution of 5000 simulated differences. (iii) The observed difference was then compared to this null distribution; if it fell outside the 95% confidence interval (i.e., *p* < 0.05), the two trajectories were considered significantly different. This non-parametric approach makes no assumptions about the data distribution and is suitable for the relatively short time series in this study.

#### 3.5.4. Diagnostic Criteria for Developmental Patterns

To systematically identify specific developmental patterns beyond the general trends addressed in the research questions, the following criteria were established prior to data analysis, drawing on previous DST studies (e.g., Larsen-Freeman, 2006; Li, 2011) and the characteristics of the current dataset (e.g., the observed range of values). (1) Retrogression was identified when an index value showed a sustained decrease over at least two consecutive time points following a period of increase. (2) Attractor state was defined as a period of relative stability in which index values fluctuated within a narrow range (e.g., ±5% of the mean) or remained stable across at least two consecutive tasks. (3) Fossilization was considered to have occurred when an index exhibited no net change across at least two consecutive time points during the observation period. (4) Trade-off (competitive) relationship was identified when two indices showed opposite directions of change (one increases while the other decreases) for at least half of the observation period (i.e., ≥8 out of 16 time points), provided that the two indices are not definitionally or mathematically interdependent. (5) Supportive relationship was identified when two indices showed the same direction of change (both increase or both decrease) for at least half of the observation period (i.e., ≥8 out of 16 time points), provided that the two indices are not definitionally or mathematically interdependent. (6) Structural coupling was identified when two indices were definitionally or mathematically interdependent (e.g., zero components necessarily occur within topic chains). In such cases, parallel development is expected and should not be interpreted as indicative of a supportive relationship. Instead, the theoretically meaningful aspect is the stability of their ratio over time. When structural coupling is present, the criteria for classifying relationships as supportive or competitive relationships do not apply. Correlation estimates for such pairs are inflated by definition and are not interpreted as evidence of cognitive coupling.

## 4. Results

In this section, the descriptions of developmental patterns (e.g., peaks, troughs, and fluctuations) are based on visual inspection of group-level and individual-level trajectories. Formal time-series analyses are beyond the scope of this exploratory study but are recommended for future research.

### 4.1. Changes in Syntactic Indices

#### 4.1.1. Changes in the Number of Topic Chains

**Figure 2:**
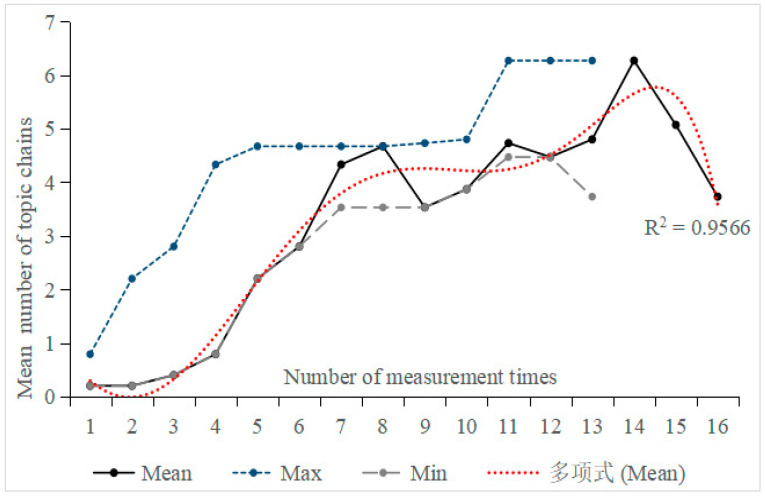
Trend of the mean number of topic chains across 15 participants over 16 tasks. The Chinese term “多项式” appearing in the legends refers to a sixth-degree polynomial.

In Figure 2, the fitting result of a sixth-degree polynomial^2^ indicated an increase in the number of topic chains, especially during the first half (see Figure 2). Regarding the mean values, dynamic fluctuations were observed, with notably higher values during the thirteenth to fifteenth measurements (M_13_ = 4.80; M_14_ = 6.27; M_15_ = 5.07). Although these tasks yielded higher values, teacher-rated difficulty did not differ significantly across tasks (see Section 3.2), suggesting that the observed fluctuations are unlikely to be driven solely by variation in task difficulty. Furthermore, the difference between the minimum and maximum values of the number of topic chains exhibited continuous variation.

**Figure 3:**
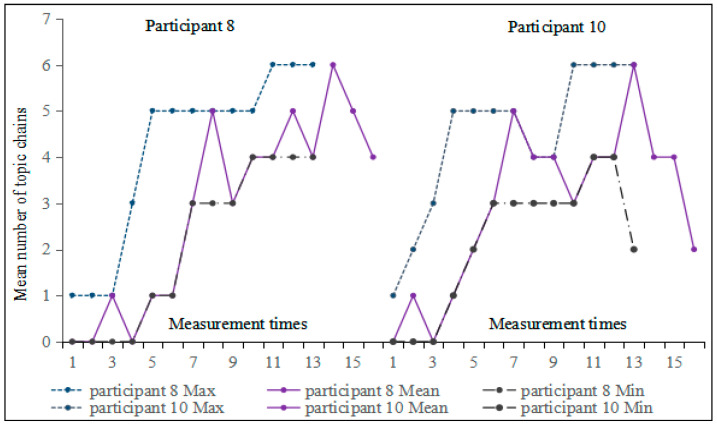
Moving min-max graphs of the number of topic chains for Participants 8 and 10 across 16 tasks.

The main focus of Figure 2 is to illustrate the overall developmental trend in the number of topic chains. However, it should be noted that not all participants conform to this trend. Variations were observed in the developmental trajectories of the number of topic chains among the 15 participants, highlighting the significant role of individual differences in L2 acquisition research (Wen, 2009). Therefore, to examine individual differences, this study calculated the correlation between measurement times and the number of topic chains, and selected the participants with the highest correlation coefficient (Participant 8: r = 0.883, *p* < 0.05) and the lowest correlation coefficient (Participant 10: r = 0.667, *p* < 0.05)^3^. Subsequently, moving min-max graphs were drawn for further analysis (see Figure 3).

The number of topic chains in compositions by Participant 8, as depicted in Figure 3, showed a fluctuating upward trend. Moreover, the difference between the minimum and maximum values (referred to here as the extremum bandwidth) also reflects instability. A relatively large extremum bandwidth occurred in the fourth to sixth moving subseries. As for Participant 10, the number of topic chains increased continuously from the third to the seventh writing tasks, but it alternated between rising and falling starting from the eighth task. The largest difference between the minimum and maximum values occurred in both the fourth and thirteenth moving windows, with a value of 4. Additionally, we conducted a Monte Carlo simulation to examine changes in the number of topic chains for these two participants. The results indicated no significant change in the number of topic chains for Participant 8 (*p* = 0.129 > 0.05, Cohen’s d = 0.23) or Participant 10 (*p* = 0.087 > 0.05, Cohen’s d = 0.31). The small effect sizes indicate that any observed changes were modest in magnitude; however, the non-significant results may be partly attributable to limited statistical power.

#### 4.1.2. Changes in the Length of Topic Chain Clauses

**Figure 4:**
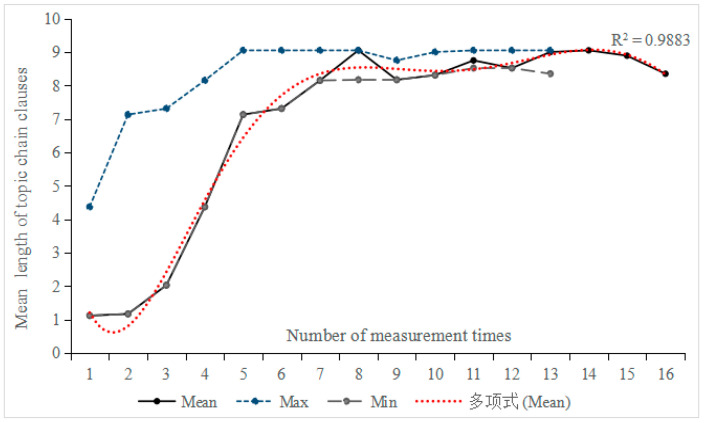
Trend of the mean length of topic chain clauses across 15 participants over 16 tasks. The Chinese term “多项式” appearing in the legends refers to a sixth-degree polynomial.

As shown in Figure 4, the development of the length of topic chain clauses exhibited a non-linear and intricate pattern, characterized by substantial variation. From the second to the fifth measurement time points, this index showed an almost linear increase; thereafter, it developed steadily starting from the sixth measurement, peaking at 9.05 in both the eighth and thirteenth tasks. Moreover, the extremum bandwidth gradually narrowed, indicating increasing developmental stability. Based on the correlation between measurement times and the length of topic chain clauses, we selected two participants for further analysis: Participant 8 (r = 0.834, *p* < 0.05), exhibiting a high correlation, and Participant 4 (r = 0.091, *p* > 0.05), exhibiting a low correlation.

**Figure 5:**
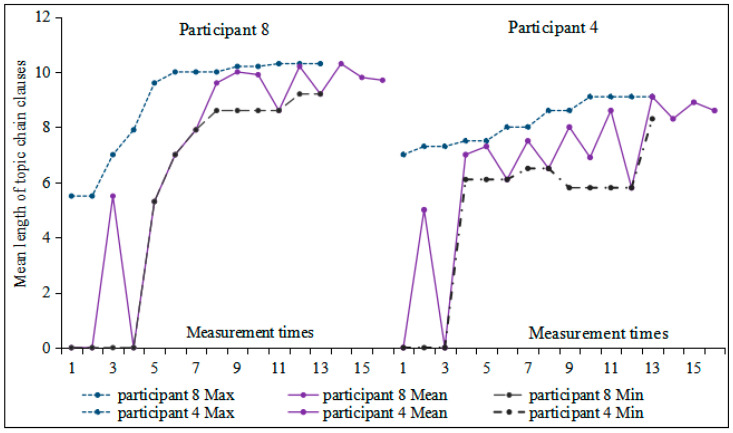
Moving min-max graphs of the length of topic chain clauses for Participants 8 and 4 across 16 tasks.

Figure 5 reveals significant changes in the length of topic chain clauses for Participants 8 and 4 across the first four tasks, followed by increased fluctuation from the fifth measurement onwards. In terms of extremum bandwidth, Participant 8 showed a gradual narrowing of the difference between minimum and maximum values after the fourth moving window, indicating relatively stable development. However, Participant 4 continued to exhibit constant changes and instability. Monte Carlo simulation indicated a significant change in the length of topic chain clauses for Participant 8 (*p* = 0.037 < 0.05, Cohen’s d = 0.52), corresponding to a medium effect size. In contrast, the change for Participant 4 remained statistically non-significant (*p* = 0.063 > 0.05, Cohen’s d = 0.41), suggesting that the observed fluctuations were smaller in magnitude.

#### 4.1.3. Changes in the Number of Topic Chain Clauses

**Figure 6:**
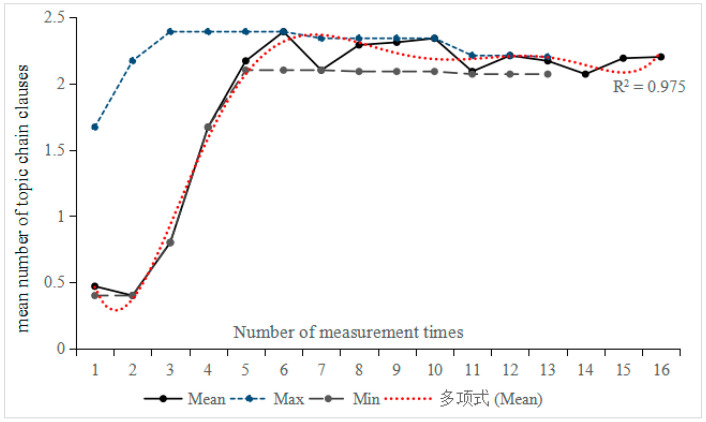
Trend of the mean number of topic chain clauses across 15 participants over 16 tasks. The Chinese term “多项式” appearing in the legends refers to a sixth-degree polynomial.

Figure 6 illustrates a general upward trend in the number of topic chain clauses, showing nearly linear growth during the first half and peaking at the sixth measurement (M = 2.39). Subsequently, a wave-like pattern emerged starting from the seventh measurement onward. Moreover, the difference between minimum and maximum values gradually diminished, indicating increasing stability. Correlation analysis between measurement time and the number of topic chain clauses revealed that Participant 12 demonstrated the highest correlation coefficient (r = 0.706, *p* < 0.05), while Participant 15 exhibited the lowest (r = 0.311, *p* > 0.05). These two participants were selected for further investigation into individual differences.

**Figure 7:**
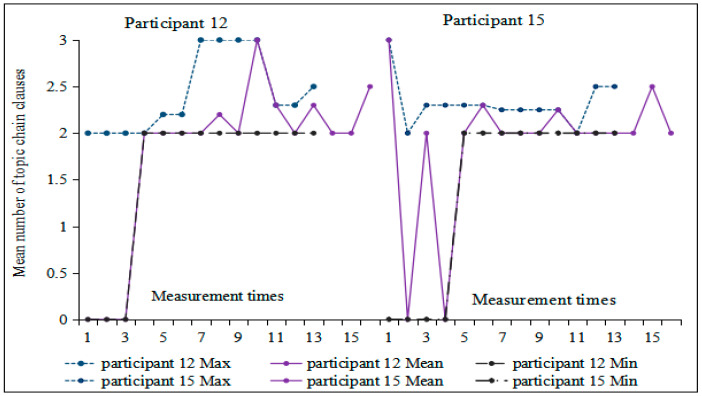
Moving min-max graphs of the number of topic chain clauses for Participants 12 and 15 across 16 tasks.

Based on Figure 7, no topic chain clauses appeared in any of the first three compositions by Participant 12. Starting from the fourth composition, the number of topic-chain clauses showed an overall upward trend, albeit with some fluctuations. In contrast, Participant 15 exhibited substantial variation in this index across the first five measurements; from the sixth measurement onward, the amplitude of variation gradually decreased. Furthermore, there were relatively large differences in the minimum and maximum values within the first three moving windows of both participants. The extremum bandwidth of Participant 15 decreased from the fifth moving window, while it continued to fluctuate for Participant 12 but with a decreasing amplitude. Monte Carlo simulation indicated that the number of topic chain clauses for Participants 12 and 15 underwent a significant change (Participant 12: *p* = 0.034 < 0.05, Cohen’s d = 0.48; Participant 15: *p* = 0.01 < 0.05, Cohen’s d = 0.62). Visual inspection of their trajectories suggests that this change may reflect a transition into a relatively stable developmental phase.

#### 4.1.4. Changes in the Number of Zero Components

**Figure 8:**
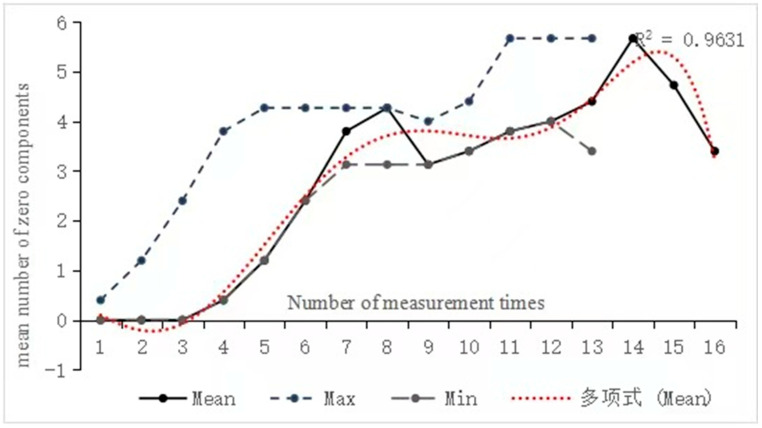
Trend of the mean number of zero components across 15 participants over 16 tasks. The Chinese term “多项式” appearing in the legends refers to a sixth-degree polynomial.

Figure 8 shows an undulating growth trend in the number of zero components, with a peak observed at the fourteenth measurement (M = 5.67). Additionally, the extremum bandwidth gradually narrowed, indicating progressive stabilization over time. When examining the correlation between the number of zero components and measurement times, Participant 8 exhibited the highest correlation coefficient (r = 0.904, *p* < 0.05), while Participant 12 demonstrated the lowest (r = 0.676, *p* < 0.05). Then we conducted an analysis on individual differences.

**Figure 9:**
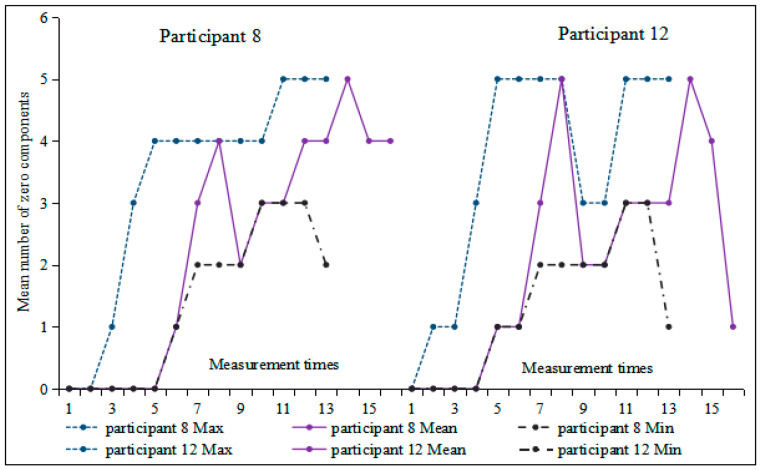
Moving min-max graphs of the number of zero components for Participants 8 and 12 across 16 tasks.

The number of zero components for Participants 8 and 12 in Figure 9 clearly deviated from the overall trend, exhibiting distinct individual characteristics—namely, instability in the difference between minimum and maximum values. Specifically, Participant 12 experienced significant fluctuations in the number of zero components, with severe drops observed at the ninth and sixteenth measurement time points, while Participant 8 exhibited only one notable drop at the ninth measurement time. Both participants continued to exhibit fluctuations in extremum bandwidth. Monte Carlo simulation indicated that neither participant showed a significant change in the number of zero components (Participant 8: *p* = 0.164 > 0.05, Cohen’s d = 0.21; Participant 12: *p* = 0.270 > 0.05, Cohen’s d = 0.18). The non-significant *p*-values and small effect sizes suggest that the observed changes were minimal in magnitude.

In summary, the above analysis reveals a general upward trend in the four indices of Chinese syntactic complexity, accompanied by frequent fluctuations. This fully demonstrates the non-linear and dynamic nature of L2 syntax development.

### 4.2. Relationship Among Syntactic Indices

#### 4.2.1. Correlation Among Syntactic Indices

The overall correlation analysis of syntactic complexity indices reveals significant correlations (r > 0.7, *p* < 0.01) among all pairwise combinations of indices^4^. It should be noted that the high correlation between ntc and nzc (r = 0.994) is partially inflated by construct overlap, as zero components necessarily occur within topic chains. Therefore, this correlation does not independently indicate a supportive relationship. Residualization checks (controlling for ntc) yielded consistent patterns, supporting the treatment of these two constructs as distinct within the DST framework. As established in Section 3.5.4, we treat this pair as structural coupling and focus on the stability of their ratio over time, rather than interpreting the raw correlation as evidence of a supportive interaction.

**Figure 10:**
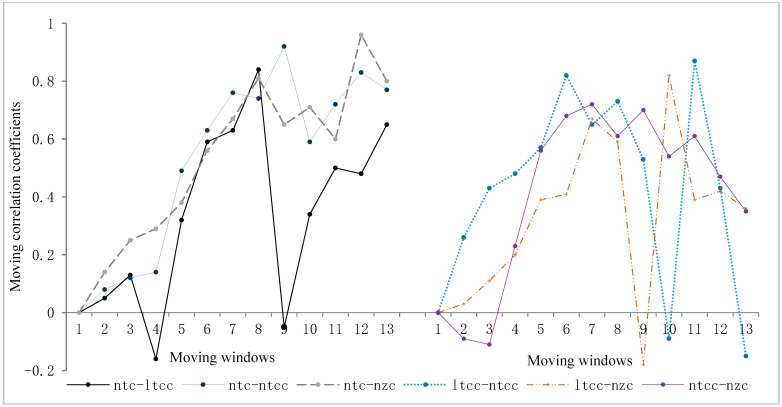
Moving correlation coefficients between all pairs of syntactic complexity indices across 16 tasks.

To further investigate the dynamic characteristics and phase-specific changes in these correlations, this study presents a graph illustrating the moving correlation coefficient (see Figure 10).

The correlation between any two syntactic complexity indices, as shown in Figure 10, did not follow a fixed or linearly increasing pattern; instead, it fluctuated constantly. Taking two groups as examples, in group 1, the correlation coefficient between the number of topic chains (ntc) and the length of topic chain clauses (ltcc) changed continually. It reached its peak at the eighth moving window (r = 0.84), while hitting its lowest point at the fourth moving window (r = −0.16). In contrast, there was considerable uncertainty in group 2 regarding the correlation between the number of topic chain clauses (ntcc) and the number of zero components (nzc). The maximum correlation coefficient observed was 0.72 in the seventh moving window, and the minimum value of −0.11 occurred in the third moving window. Thus, the relationships among syntactic complexity indices exhibited fluctuation over time rather than sustained strong correlations. Additionally, a moving correlation coefficient plot can better illustrate the stage-wise variations in correlations between any two indices.

#### 4.2.2. Interactions Among Syntactic Indices

**Figure 11:**
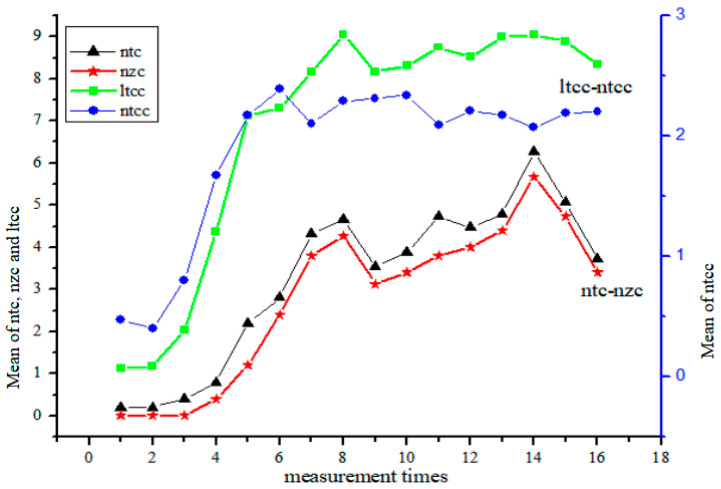
Interactive modes of the syntactic complexity indices^5^.

Constrained by text length and space limitations, this paper primarily focuses on two sets of indices to examine their interactive relationships. One set involves the number of topic chains and the number of zero components (ntc-nzc); the other set concerns the length of topic chain clauses and the number of topic chain clauses (ltcc-ntcc). Please refer to Figure 11.

Figure 11 presents the interaction between the number of topic chains (ntc) and the number of zero components (nzc), illustrating their developmental trends across tasks. A visual inspection suggests a degree of concordance, particularly during tasks 3 to 11 and 13 to 16. According to the diagnostic criteria in Section 3.5.4, ntc and nzc are definitionally interdependent. Therefore, their parallel development is treated as structural coupling rather than a supportive relationship. Monte Carlo simulation revealed no significant difference between the developmental trajectories of these two indices (*p* = 0.086 > 0.05, Cohen’s d = 0.31). This pattern warrants further investigation in future studies employing more targeted designs.

The interactive relationship between the length of topic chain clauses (ltcc) and the number of topic chain clauses (ntcc) was examined against the diagnostic criteria defined in Section 3.5.4. Across the 16 time points, the two indices showed opposite directions in 9 out of 16 comparisons (56.25%), exceeding the 50% threshold for a trade-off relationship. This pattern was particularly evident from the seventh to the sixteenth measurement, where peaks in ltcc often coincided with troughs in ntcc (e.g., at measurements 11, 12, and 14). Accordingly, the relationship between ltcc and ntcc is identified as a competitive (trade-off) pattern. The Monte Carlo simulation further indicated a significant difference between the two trajectories (*p* = 0.039 < 0.05, Cohen’s d = 0.65), supporting the robustness of this competitive interaction.

To summarize, the development of each syntactic complexity index is characterized by non-linearity and interdependence, exhibiting continuous fluctuations as well as interactions, including competitive relationships (e.g., between ltcc and ntcc) and structural coupling (e.g., between ntc and nzc). These phenomena collectively manifest the dynamic nature of syntactic complexity.

## 5. Discussions

### 5.1. Characteristics of Syntactic Development

Based on DST, this study primarily examines the diachronic features of syntactic complexity in CSL writings by 15 Cambodian native speakers. The findings are as follows. (i) The indices of syntactic complexity generally showed an upward trend over time, albeit with noticeable nonlinearity and turbulence. (ii) The syntactic complexity indices were closely interrelated, exhibiting different forms of connection and interaction. (iii) Individual differences in syntactic acquisition were observed, with learners following unique developmental trajectories rather than converging on a common one.

According to the results mentioned above, syntactic development patterns can be categorized into two types. The first type shows an increasing trend in syntactic indices, with the growth rate initially high and gradually stabilizing over time. For instance, the number of topic chains increased rapidly in the first half, reaching a peak amplitude of 1.53; in contrast, growth slowed in the second half, with a maximum increment of 1.47. Similarly, the number of zero components also grew slowly in the second half and gradually stabilized. However, these results are inconsistent with Wu’s (2017) empirical findings that both the number of topic chains and the number of zero components maintained substantial growth during the mid- to late stage. The two empirical results may differ for two potential reasons: one may be related to variations in L2 learners’ mother tongues, and another may be attributed to their distinct levels of Chinese language cognition. The participants in this study are 15 Cambodian native speakers. Based on language typology, it has been suggested that the Cambodian language places equal emphasis on both topic and subject, while the Chinese language prioritizes the topic by emphasizing discourse. This typological proximity may explain the developmental patterns observed. As CSL learners progress to intermediate and advanced levels, their comprehension of the distinctions between Chinese and Cambodian languages may gradually deepen and stabilize, which could account for the slight increase in the number of topic chains and number of zero components. However, as the current study did not directly measure typological distance or learners’ cognitive development, this interpretation remains preliminary. Future research employing targeted assessments is needed to validate these explanations.

The second type involves a decrease in syntactic indices. Retrogression, an inevitable and unpredictable aspect of knowledge development (Larsen-Freeman, 2006), reflects the self-organization and self-adaptability of language (Larsen-Freeman & Cameron, 2008). In this study, retrogression was observed across multiple syntactic indices. For example, the length of topic chain clauses declined during tasks 11 to 12 and 14 to 16. Furthermore, there was a decrease in the number of zero components from the eighth to the ninth tasks, as well as from the fourteenth to the sixteenth measurements. These changes can be attributed to unstable Chinese language proficiency among L2 learners, which leads to repeated fluctuations in their acquisition levels and continuous self-organization and reconstruction of syntactic knowledge. Consequently, this triggers the emergence of pre-existing linguistic knowledge (Van Geert, 2003), ultimately resulting in linguistic retrogression.

In addition, during syntactic development, a phenomenon known as fossilization can occur, which is referred to as the attractor state within DST (Li, 2011). For example, attractor states were observed in the number of zero components of Participant 12 during tasks 5 to 6, 9 to 10, and 12 to 13. Such states represent temporary stagnation rather than a final developmental endpoint. In fact, language acquisition involves transitioning from one attractor state to another while encountering various challenges and experiencing different degrees of growth or attrition at multiple levels. Thus, progress and regression coexist in language learning, and fossilization has no definitive endpoint (Zheng, 2025).

### 5.2. Interactive Relationship of Syntactic Indices

There are interactive relationships among language subsystems, forming connected growers (Van Geert, 2008). Supportive relationships occur when indices increase or decrease simultaneously, whereas competitive relationships arise when one index falls while another rises (Li, 2011). In terms of the syntactic subsystem, the interactive relationships among indices are neither singular nor fixed, but involve a dynamic coexistence of mutual support and competition that varies over time (Chan et al., 2015).

#### 5.2.1. Competitive Interaction

Following the diagnostic criteria in Section 3.5.4, the competitive pattern between ltcc and ntcc (9/16 opposite changes) reflects a trade-off relationship, consistent with Skehan’s (1998) Trade-off Hypothesis. The Trade-off Hypothesis posits that, due to limited human attentional capacity and working memory, individuals cannot simultaneously allocate their limited attention and cognitive resources evenly across multiple language subsystems, resulting in inevitable competition. We selected the length of topic chain clauses and the number of topic chain clauses for further analysis. When cognitive resources are sufficient, the length of topic chain clauses steadily increases, peaking at the eighth and fourteenth tasks, whereas the number of topic chain clauses is at its lowest during the same period. The reason is that when learners focus their attention on one aspect of syntactic complexity, their attention naturally decreases for others. However, once the length of topic chain clauses reaches its peak, indicating that no additional resources are needed to maintain optimal performance (Johnson et al., 2012), more resources can be allocated to the connected grower. Therefore, when the length of topic chain clauses reaches its peak, this point can be considered the inflection point at which the number of topic chain clauses begins to increase. Consequently, in a competitive relationship, an increase in one index is conditional upon a decrease in the other, with the two indices mutually constraining each other and resulting in crests that correspond to troughs. Actually, the competitive interaction between the length of topic chain clauses and the number of topic chain clauses primarily reflects learners’ mastery of linguistic knowledge and their inertia in application, rather than inherent properties of language itself. This embodies the self-organization and self-adaptation characteristics of the language system.

#### 5.2.2. Structural Coupling Between Topic Chains and Zero Components

As established in the diagnostic criteria (Section 3.5.4), the number of topic chains (ntc) and the number of zero components (nzc) are definitionally interdependent because zero components necessarily occur within topic chains. Therefore, their relationship is identified as structural coupling rather than a supportive or competitive interaction as defined by Li (2011). Given this definitional dependency, the parallel developmental trajectories observed in Figure 11 are expected and do not, by themselves, provide evidence of cognitive support. What is theoretically meaningful, however, is the stability of their ratio over time. The high raw correlation (r = 0.994) is expected given this definitional dependency and is not interpreted as evidence of cognitive support. Despite fluctuations in absolute values across the 16 tasks, ntc and nzc maintained a consistent proportional relationship throughout the observation period (see Figure 11). This stability suggests that learners’ discourse organization remained internally coherent, even as individual indices varied.

The Monte Carlo simulation further indicated no significant difference between the two developmental trajectories (*p* = 0.086 > 0.05; Cohen’s d = 0.31), which is consistent with the interpretation of structural coupling. Future research should examine whether this structural coupling holds across different task types and proficiency levels, and whether it can be disrupted by targeted instructional interventions.

## 6. Conclusions

This study analyzes the changing trends and potential interactions of syntactic complexity indices in CSL compositions within the framework of DST. The results reveal features such as jumps, variability, dynamicity, and non-linearity in the syntactic development of CSL learners. Several pedagogical implications are proposed.

Firstly, the indices of Chinese syntactic complexity fluctuate, reflecting both progress and regression. Therefore, researchers may benefit from adopting a DST perspective to move beyond static research paradigms and engage more directly with the complex and variable nature of language acquisition. Future work could focus on dynamic changes within the syntactic system and explore both internal mechanisms and external constraints to deepen our understanding of syntax acquisition. Additionally, although group-level patterns are informative, greater attention could be paid to modeling individual developmental trajectories.

Secondly, the indices of syntactic complexity do not develop independently but rather interact with one another. One result is that the number of topic chains and the number of zero components generally develop synchronously. This suggests that, in CSL writing instruction, teachers might consider introducing the concepts of topic chains and zero components together, providing sample texts, and encouraging students to imitate authentic expressions. Such practice may help learners internalize these features and increase their use of topic chains and zero components.

Thirdly, the development of Chinese syntax is characterized by multidimensionality and uniqueness. This highlights the importance of constructing a comprehensive measurement system for Chinese syntax, including topic chain, zero component, and others. Future research could also explore additional indices to capture the full range of Chinese syntactic complexity.

In addition, several limitations should be acknowledged. First, despite acceptable inter-rater reliability (Cohen’s κ ranged from 0.78 to 0.84), the teacher-as-researcher role may have introduced bias. Future research should involve multiple coders blind to the hypotheses and separate instructional from research roles. Second, the small sample (n = 15) and single-institution setting limit both statistical power and generalizability. Future research should include larger and more diverse samples from multiple institutions. Third, although teacher-rated difficulty was comparable across tasks, topic content still varied, so observed fluctuations may partly reflect task effects. Task difficulty is only a partial proxy; content-specific features (e.g., concrete vs. abstract) may still affect syntactic choices. Future research should employ more controlled designs, such as using repeated prompts or statistically controlling for task difficulty. Fourth, some indices may be mathematically related by definition. For example, zero components occur within topic chains, so a higher number of topic chains mechanically allows for a higher number of zero components. This structural dependency may inflate observed correlations. Future research should normalize frequency-based indices (e.g., per 100 clauses) to address this issue. Moreover, future research should include a wide range of writing genres, examine how Chinese syntactic complexity changes across different genres, and explore the triggering mechanism and constraints associated with individual differences.

## Figures and Tables

**Table 1:** Research materials contributed by all participants.

	Topics	Number (Writings)	Average (Characters)	Total (Characters)
Semester I	family, hobby, friendship, job, food, interest, gift, campus life.	120	349	41,880
Semester II	dress, travel, dream, entertainment, change, favorite books, unforgettable experience, challenge	120	462	55,440
Total	16	240	405.5	97,320

**Table 2:** Examples of coding decisions for topic chains and zero components.

Examples	Coding Decisions	Explanations
(1) Participant 7, task 2 我喜欢旅游，ø喜欢跑步，ø喜欢爬山，ø喜欢跟朋友一起吃饭。 Wo xihuan lvyou, ø xihuan paobu, ø xihuan pashan, ø xihuan gen pengyou yiqi chi fan.	Topic chain 我喜欢旅游 → ø喜欢跑步 → ø喜欢爬山 → ø喜欢跟朋友一起吃饭	The four clauses share the same topic “wo (我)”, forming a topic chain. The subject “wo (我)” is omitted in all three instances of ø, and it can be clearly restored from the first clause, meeting the criteria for counting zero components.
	Zero components ø喜欢跑步 ø喜欢爬山 ø喜欢跟朋友一起吃饭	
(2) Participant 12, task 1 在我的心中，爸爸是一个大英雄，ø可以保护妈妈和我，ø可以给我想要的东西，我很爱我的爸爸。 Zai wo de xin zhong, baba shi yi ge da yingxiong, ø keyi baohu mama he wo, ø keyi gei wo xiang yao de dongxi, wo hen ai wo de baba.	Topic chain 1 爸爸是一个大英雄 → ø可以保护妈妈和我 → ø可以给我想要的东西 Topic chain 2 我很爱我的爸爸	The first three clauses share the topic “baba (爸爸)”, forming a topic chain. The subject of the last clause changes to “wo (我)”, starting a new chain. Both instances of ø omit the subject “baba (爸爸)”, which can be clearly restored from the context and should be counted as zero components.
	Zero components ø可以保护妈妈和我 ø可以给我想要的东西	
(3) Participant 3, task 7 姐姐送我一个礼物，ø是一条裙子，ø很漂亮，我穿一下，但是妈妈觉得不好看，ø不喜欢，后来就退了裙子呢。 Jiejie song wo yi ge liwu, ø yi tiao qunzi, ø hen piaoliang, wo chuan yi xia, danshi mama jue de bu hao kan, ø bu xihuan, houlai jiu tui le qunzi ne.	Topic Chain 1 姐姐送我一个礼物 → ø是裙子 → ø很漂亮 Topic Chain 2 我试穿一下 Topic Chain 3 妈妈觉得不好看 → ø不喜欢 (The sentence “后来就退了裙子呢” cannot be assigned to any topic chain because its subject is indeterminate.)	The first three clauses share the topic “liwu (礼物)” and form a topic chain. The clause “我穿一下” shifts the subject and initiates a new chain. “但是妈妈觉得不好看” and “ø不喜欢” share the topic “mama (妈妈)” and form the third topic chain. The subject of the last clause (后来就退了裙子呢) is missing and cannot be clearly recovered. This is a learner error and is not counted as a zero component.
	Zero components ø是裙子; ø很漂亮; ø不喜欢	

## Data Availability

The data presented in this study are available on request from the corresponding author due to privacy reasons.

## Notes

1 Other contextual factors (e.g., socioeconomic status, first language literacy, media exposure, tutoring) were not measured and may contribute to individual variability. Future research should document these variables.

2 A sixth-degree polynomial was fitted for descriptive purposes to smooth short-term fluctuations. Lower-degree polynomials underfitted the observed patterns, whereas higher-degree polynomials did not improve the fit (adjusted R2). The sixth-degree polynomial was selected as the lowest order that captured the main inflection points without overfitting, as indicated by cross-validated prediction error. Although higher R2 values may also reflect overfitting to this specific sample, the precise shape of the trajectories should be interpreted with caution until replicated in larger, independent samples. This fitting is purely descriptive and does not imply that the underlying developmental process follows a sixth-degree polynomial function.

3 Two extreme cases, those with the highest and lowest correlations, were selected for exploratory illustration. However, this approach may exaggerate contrasts and does not represent the full sample. Future research should use multilevel models to analyze data from all participants.

4 The four indices are abbreviated as follows: the number of topic chains (ntc), the length of topic chain clauses (ltcc), the number of topic chain clauses (ntcc), and the number of zero components (nzc). The overall correlation results between any two indices are: r(ntc − ltcc) = 0.934, r(ntc − ntcc) = 0.793; r(ntc − nzc) = 0.994; r(ltcc − ntcc) = 0.952; r(ltcc − nzc) = 0.914; r(ntcc − nzc) = 0.768.

5 Note that the dual y-axes in Figure 11 have different scales. The left y-axis corresponds to the ntc, nzc and ltcc, while the right y-axis to the ntcc. And our interpretation focuses on the direction and shape of the trajectories rather than a direct comparison of their absolute magnitudes.
